# Molecular surveillance of *Plasmodium falciparum* drug resistance in the Republic of Congo: four and nine years after the introduction of artemisinin-based combination therapy

**DOI:** 10.1186/s12936-017-1816-x

**Published:** 2017-04-19

**Authors:** Felix Koukouikila-Koussounda, Sankarganesh Jeyaraj, Christian N. Nguetse, Charles Nchotebah Nkonganyi, Kossiwa Clarisse Kokou, Mandingha K. Etoka-Beka, Francine Ntoumi, Thirumalaisamy P. Velavan

**Affiliations:** 1 Fondation Congolaise pour la Recherche Médicale (FCRM), Brazzaville, Republic of Congo; 2 Institute of Tropical Medicine, University of Tübingen, Tübingen, Germany; 3 Duy Tan University, Da Nang, Vietnam; 4 Vietnamese-German Centre for Medical Research, Hanoi, Vietnam

**Keywords:** Malaria, *Plasmodium falciparum*, *Pfcrt*, *Pfmdr1*, *Pfatp6*, *Pfk13*, Anti-malarial resistance, ACT, Republic of Congo

## Abstract

**Background:**

Resistance to anti-malarial drugs hinders efforts on malaria elimination and eradication. Following the global spread of chloroquine-resistant parasites, the Republic of Congo adopted artemisinin-based combination therapy (ACT) in 2006 as a first-line treatment for uncomplicated malaria. To assess the impacts after implementation of ACT, a molecular surveillance for anti-malarial drug resistance was conducted in Congo 4 and 9 years after the introduction of ACT.

**Methods:**

Blood samples of 431 febrile children aged 1–10 years were utilized from two previous studies conducted in 2010 (N = 311) and 2015 (N = 120). All samples were screened for malaria parasites using nested PCR. Direct sequencing was used to determine the frequency distribution of genetic variants in the anti-malarial drug-resistant *Plasmodium falciparum* genes (*Pfcrt, Pfmdr1*, *Pfatp6, Pfk13*) in malaria-positive isolates.

**Results:**

One-hundred and nineteen (N = 70 from 2010 and N = 49 from 2015) samples were positive for *P. falciparum*. A relative decrease in the proportion of chloroquine-resistant haplotype (CVIET) from 100% in 2005, 1 year before the introduction and implementation of ACT in 2006, to 98% in 2010 to 71% in 2015 was observed. Regarding the multidrug transporter gene, a considerable reduction in the frequency of the mutations N86Y (from 73 to 27%) and D1246Y (from 22 to 0%) was observed. However, the prevalence of the Y184F mutation remained stable (49% in 2010 compared to 54% in 2015). Isolates carrying the *Pfatp6* H243Y was 25% in 2010 and this frequency was reduced to null in 2015. None of the parasites harboured the *Pfk13* mutations associated with prolonged artemisinin clearance in Southeast Asia. Nevertheless, 13 new *Pfk13* variants are reported among the investigated isolates.

**Conclusion:**

The implementation of ACT has led to the decline in prevalence of chloroquine-resistant parasites in the Republic of Congo. However, the constant prevalence of the *PfMDR1* Y184F mutation, associated with lumefantrine susceptibility, indicate a selective drug pressure still exists. Taken together, this study could serve as the basis for epidemiological studies monitoring the distribution of molecular markers of artemisinin resistance in the Republic of Congo.

## Background

In the Republic of Congo, malaria remains one of the leading causes of morbidity and mortality. Chloroquine and sulfadoxine–pyrimethamine were extensively used for several decades as first-line treatment for uncomplicated malaria, but parasites rapidly developed resistance to both these drugs [1–5]. As a result, in 2006, Congo changed its national drug policy and switched to artesunate + amodiaquine (ASAQ) and artemether + lumefantrine (AL) combinations as first-line and second-line drugs, respectively, for the treatment of acute uncomplicated malaria.

Chloroquine resistance (CQR) is caused by mutations in two genes, *Plasmodium falciparum* chloroquine resistance transporter (*Pfcrt*) and multidrug resistance transporter-1 (*Pfmdr1*), both located on the digestive food vacuole of the parasite [6]. These two genes also reduce the susceptibility to other quinolone anti-malarial agents such as amodiaquine, lumefantrine and mefloquine [7–9]. Many studies reported that in the absence of drug pressure, chloroquine-resistant strains have been replaced by sensitive ones [10, 11].

Many *P. falciparum* candidate genes are believed to be associated with artemisinin resistance. The endoplasmic and sarcoplasmic reticulum Ca^2+^-ATPase orthologue of *P. falciparum* (*Pfatp6*) has been postulated to be the target of artemisinin [12]. The H243Y, L263E, E431K, A623E, and S769N mutations have been shown to influence the functional activities in *Pfatp6* [13–15]. In 2014, Ariey et al. showed that mutations (C580Y, R539T, Y493H, M476I) in the *Pfk13* gene were associated with delayed parasite clearance [16]. As chloroquine-resistant *P. falciparum* strains spread worldwide from Southeast Asia, a similar devastating effect can also occur in case of artemisinin-resistant isolates. However, it has been shown that artemisinin-resistant parasites could also emerge independently in Southeast Asia, as *Pfk13* mutations occur unexpectedly in many sites within the region [17].

Long-term monitoring of parasite sensitivity to previously withdrawn anti-malarial drugs, such as CQ, can provide useful surveillance information if these drugs target similar resistance markers to current or candidate ACT partner drugs [18]. Therefore, the aim of this study was to determine the prevalence of polymorphisms in the anti-malarial resistance genes, including *Pfcrt*, *Pfmdr1*, *Pfatp6*, and *Pfk13* in field isolates from the Republic of Congo collected from two cross-sectional studies conducted in 2010 and 2015, approximately four and nine years after the adoption of ACT.

## Methods

### Study site and sample collection

The study was conducted in the Southern and Northern districts in Brazzaville, the capital of the Republic of Congo. Malaria occurs holo-endemically and the transmission rates in the country are high and perennial, the majority of malaria cases being caused by *P. falciparum*. A total of 431 children aged 1–9 years were recruited in two phases: from April to June 2010 [Group A (N = 311)] and from September 2014 to February 2015 [Group B (N = 120)] from previous studies [19, 20] were utilized in this study. The 2010 cohort was from three districts of Makélékélé health division in southern region of Brazzaville, Republic of Congo. This cohort comprised of children aged 1–9 years and are permanent residents of the study area. The second cohort (September 2014 to February 2015) was collected from children aged from 1 to 10 years presenting with fever at the paediatric ward of the MNG hospital in Talangai, a northern Brazzaville region in Republic of Congo. This study was conducted in febrile children presenting with fever at the MNG hospital. Children who were positive for malaria by microscopy were included. Febrile children with other pathologies such as severe diarrhoea, pulmonary infection and HIV were excluded. At the time of enrolment 4 mL of whole blood were collected in heparinized tubes from all children and thick and thin blood smears were performed. In the case of malaria (positive thick and thin blood smears with axillary temperature ≥37.5 °C), collected blood samples were stored at −80 °C for molecular analyses, parasite density determined and children were treated with AS + AQ or AL. Ethical approval was given by the Institutional Ethics Committee for Research on Health Sciences of the Republic of Congo. Written informed consent was obtained from parents or guardians of children.

### *Plasmodium* species identification, and *Pfcrt*, *Pfmdr1*, *Pfatp6*, *Pfk13* genotyping

Genomic DNA was extracted from 200 μL of peripheral whole blood samples using QIAamp DNA Blood Mini Kit (Qiagen, Hilden, Germany). The *Plasmodium* 18S rRNA gene was amplified by nested PCR to detect positive samples and further differentiate the species as described elsewhere [21, 22]. The primer pairs and thermocycling conditions are summarized in Table 1. Fragments of the *Pfcrt* (410 bp containing the polymorphisms at codons 72–76), *Pfmdr1* [(580 bp including the polymorphisms at codons 86 and 184) and (864 bp containing the polymorphisms at codons 1034–1246)], *Pfatp6* (800 bp carrying the polymorphisms at codons 243–769) and *Pfk13* (849 bp comprising the main polymorphisms associated with delayed clearance in Southeast Asia) were amplified by nested PCR as described elsewhere [11, 23, 24]. The primer sequences and PCR conditions are described in Table 2. The PCR products were visualized through electrophoresis on 1.2% agarose gel stained with SYBR Green I in 1× Tris-electrophoresis buffer (90 mM Tris–acetate, pH 8.0, 90 mM boric acid, 2.5 mM EDTA). Thereafter, PCR products were purified using Exo-SAP-IT (USB, Affymetrix, USA) and directly used as templates for DNA sequencing using the BigDye terminator v. 1.1 cycle sequencing kit (Applied Biosystems, Foster City, USA) on an ABI 3130XL DNA sequencer. Single nucleotide polymorphisms (SNPs) were identified by assembling the sequences with each reference sequence using Codon code Aligner 4.0 software and were reconfirmed visually from their respective electropherograms.

**Table 1:** Primer sequences and cycling conditions for *Plasmodium* species identification

Genus and species	Primer name	Primer sequence (5′-3′)	PCR conditions	Reference
*Plasmodium genus*	rPLU 1 rPLU 5	TCAAAGATTAAGCCATGCAAGTGA CCTGTTGTTGCCTTAAACTTC	95 °C for 5 min; 25× [94 °C for 1 min; 58 °C for 1 min; and 72 °C for 2 min]; 72 °C for 5 min	Snounou et al. [22]
*P. falciparum*	rFAL1 rFAL2	TTAAACTGGTTTGGGAAAACCAAATATATT ACACAATGAACTCAATCATGACTACCCGTC	95 °C for 5 min; 30× [94 °Cfor 1 min; 58 °C for 1 min; and 72 °C for 2 min]; 72 °C for 5 min	
*P. malariae*	rMAL1 rMAL2	ATAACATAGTTGTACGTTAAGAATAACCGC AAAATTCCCATGCATAAAAAATTATACAAA		
*P. vivax*	rVIV1 rVIV2	CGACTTCCAAGCCGAAGCAAAGAAAG TCCTTACTTCTAGCTTAATCCACATAACTGATAC		
*P. ovale*	rOVA1WC rOVA2WC	TGTAGTATTCAAACGCAGT TATGTACTTGTTAAGCCTTT		Fuehrer et al. [21]

**Table 2:** Primers and PCR conditions for the amplification of different drug resistance loci

Loci	Primer name	Primer (5′-3′)	PCR conditions	Reference
*Pfcrt* SNPs at codons 72 and 76	OF P1 OR P2	5′-CCGTTAATAATAAATACACGCG-3′ 5′-CGGATGTTACAAAACTATAGTCC-3′	35 cycles of 94 °C for 30 s; 56 °C for 30 s; and 62 °C for 1 min	Mekonnen et al. [11]
	NF P3 NR P4	5′-AGGTTCTTGTCTTGGTAAATTTGC-3′ 5′-CAAAACTATAGTTACCAATTTTG-3′	30 cycles of 94 °C for 30 s; 56 °C for 30 s; and 65 °C for 1 min	
*Pfmdr1* SNPs at codon 86 and 184	OF P5 OR P6	5′-AGGTTGAAAAAGAGTTGAAC-3′ 5′-ATGACACCACAAACATAAAT-3′	30 cycles of 94 °C for 30 s; 55 °C for 30 s; and 65 °C for 1 min	
	NF P7 NR P8	5′-ACAAAAAGAGTACCGCTGAAT-3′ 5′-AAACGCAAGTAATACATAAAGTC-3′	30 cycles of 94 °C 30 s; 60 °C for 30 s; and 65 °C for 1 min	
*Pfmdr1* SNPs at codon 1034,1024, and 1246	OF P9 OR P10	5′-GTGTATTTGCTGTAAGAGCT-3′ 5′-GACATATTAAATAACATGGGTTC-3′	34 cycles of 94 °C for 30 s; 55 °C for 1 min and 72 °C for 2 min	
	NF P11 NR 12	5′-CAGATGATGAAATGTTTAAAGATC-3′ 5′-TAAATAACATGGGTTCTTGACT-3′	29 cycles of 94 °C for 30 s; 60 °C for 30 s; and 65 °C for 1 min	
*Pfatp6* at codon 243, 263 and 431	NF P13 NR P14	5′-TCATCTACCGCTATTGTATG-3′ 5′-TCCTCTTAGCACCACTCC-3′	35 cycles of 94 °C for 45 s; 55 °C for 1 min; 72 °C for 1 min	Zakeri et al. [24]
*Pfatp6* SNPs at codon 623 and 769	NF P15 NR P16	5′-TGGAGACAGTACCGAATTAGC-3′ 5′-TCTTCCTACATATTTACGTGGTG-3′		
*Pfk*13	OF P17 OR P18	5′-CGGAGTGACCAAATCTGGGA-3′ 5′-GGGAATCTGGTGGTAACAGC-3′	30 cycles of 95 °C for 30 s; 58 °C for 2 min; 72 °C for 2 min	Ariey et al. [16]
	NF P19 NR P20	5′-GCCAAGCTGCCATTCATTTG-3′ 5′-GCCTTGTTGAAAGAAGCAGA-3′	40 cycles of 95 °C for 30 s; 60 °C for 1 min; 72 °C for 1 min	

*OF* outer forward, *OR* outer reverse, *NF* nested forward, *NR* nested reverse

## Results

### *Plasmodium* species identification

Using nested PCR for the species identification, 22.5% (70/311) and 40.8% (49/120) samples collected in 2010 and 2015, respectively, were positive for *P. falciparum*. The other species namely *Plasmodium malariae*, *Plasmodium vivax* and *Plasmodium ovale* were not detected in the study samples. The positive samples for *P. falciparum* malaria infection were screened for genetic variants in the *Pfcrt*, *Pfmdr1*, *Pfatp6*, and *Pfk13* associated with anti-malarial drug resistance.

### *Pfcrt* polymorphisms

The success rate of *Pfcrt* amplicon sequencing was equally 57% with the samples from the 2010 cohort (40/70) and the 2015 cohort (28/49). The mutation frequencies are shown in Table 3. A triple mutation M74I, N75E and K76T was observed, each occurring at a frequency of 98% in 2010 and 71% in 2015. All the mutants carried the Cysteine–Valine–Isoleucine–Glutamate–Threonine (CVIET) haplotype while the wild type strains had the Cysteine–Valine–Methionine–Asparagine–Lysine (CVMNK) haplotype.

**Table 3:** Prevalence of *Pfcrt* and *Pfmdr1* polymorphisms

Locus	Mutation	Cohort 2010 (%)	Cohort 2015 (%)
*Pfcrt*	M74I, N75E and K76T	39/40 (98)	20/28 (71)
*Pfmdr1*	N86Y	30/41 (73)	7/26 (27)
	S113R	1/41 (2)	0/26 (0)
	Y184F	20/41 (49)	14/26 (54)
	T1069T	8/41 (20)	3/26 (12)
	G1113G	0/41 (0)	1/26 (4)
	D1127D	1/41 (2)	0/26 (0)
	T1157T	2/41 (5)	2/26 (8)
	V1225A	1/41 (2)	0/26 (0)
	D1246Y	9/41 (22)	0/26 (0)

Values in parentheses represent the percentage and is rounded

*N P. falciparum* positive samples

### *Pfmdr1* polymorphisms

Forty-one samples (59%) from 2010 and 26 samples (53%) from 2015 were sequenced for the detection of *Pfmdr1* polymorphisms. The frequency of the *Pfmdr1* gene mutations at positions 86 and 1246 are shown in Table 3. The frequency of N86Y mutant alleles was 73% in 2010 and 27% in 2015. The D1246Y mutation in 2010 occurred at a frequency of 22 and 0% in 2015. Neither the S1034C mutation nor the N1042D mutation in the *Pfmdr1* gene was observed in the sequenced samples. The prevalence of the Y184F variant was 49% in 2010 and 54% in 2015. Other mutations occurred at quite low frequencies are represented in Table 3.

### *Pfatp6* polymorphisms

Eight samples (11%) from 2010 and 16 samples (33%) from 2015 were successfully sequenced to identify mutations in the *Pfatp6* gene. Out of all the five key point mutations that were investigated, only H243Y and E431K mutations were observed. Both mutations occurred in 2010 at a frequency of 25% (2/8) and 12% (1/8), respectively, while in 2015, only the E431K mutation was observed occurring at a frequency of 12% (2/16). Other mutations found at positions 291 (I291V), 402 (L402V), 569 (N569K), 630 (A630S), 632 (G632E), 639 (G639D), 646 (F646F), and 747 (H747Y) are shown in Table 4. In addition to these SNPs, a variation of asparagine-tandem repeat region (codon 457–465) was observed in one sample from 2010, with an insertion of two asparagine residues after the 465th codon.

**Table 4:** Prevalence of *Pfatp6* and *Pfk13* gene polymorphisms

Locus	Mutation	Cohort 2010 (%)	Cohort 2015 (%)
*Pfatp6*	H243Y	2/8 (25)	0/16 (0)
	I291V	0/8 (0)	1/16 (6)
	L402V	1/8 (12)	2/16 (12)
	E431K	1/8 (12)	2/16 (12)
	N569K	5/8 (63)	2/16 (12)
	A630S	3/8 (38)	0/16 (0)
	G632E	0/8 (0)	1/16 (6)
	G639D	0/8 (0)	1/16 (6)
	F646F	1/8 (12)	0/16 (0)
	H747Y	0/8 (0)	1/16 (6)
*Pfk13*	D464N	1/41 (2)	0/25 (0)
	S466I	0/41 (0)	1/25 (4)
	Q467H	0/41 (0)	1/25 (4)
	N489N	0/41 (0)	1/25 (4)
	G496G	0/41 (0)	1/25 (4)
	R539R	1/41 (2)	0/25 (0)
	S549P	1/41 (2)	0/25 (0)
	I552M	0/41 (0)	1/25 (4)
	L598L	1/41 (2)	0/25 (0)
	E602E	1/41 (2)	0/25 (0)
	N609N	0/41 (0)	1/25 (4)
	F628L	0/41 (0)	1/25 (4)
	K669K	1/41 (2)	0/25 (0)

Values in parentheses represent the percentage

*N P. falciparum* positive samples

### *Pfk13* polymorphisms

Regarding the Kelch13 propeller domain, 41 out of 70 samples (59%) for 2010 cohort and 25 out of 49 samples (51%) for 2015 cohort were sequenced and used for analysis. The identified mutations in the Kelch13 propeller domain are shown in Table 4. The *Pfk13* variants M476I, Y493H, R539T, I543T, and C580Y reported to occur in Southeast Asia were not observed in the Republic of Congo. Thirteen new substitutions were identified in the *Pfk13* propeller domain. Six *Pfk*13 non-synonymous substitutions were observed in the propeller blades. The *Pfk*13 non-synonymous variants I552M and S549P observed in this study were previously reported in Central African Republic and in India, respectively.

## Discussion

Malaria is still considered as one of the major health problems in the Republic of Congo and uncomplicated malaria occurs in young children due to *P. falciparum*. Malaria incidence is 0.9 malaria episode/year/child in Brazzaville and malaria transmission is perennial both in southern and northern Brazzaville, with *P. falciparum* being the most predominant species. In this study, *P. falciparum* was the only species detected in all the malaria-positive samples. Several factors, including both malaria parasite (e.g., pyrogenic threshold, multiplication rate, cytoadhesion) and host genetics (e.g., immunity, tolerance, pregnancy, co-morbidities) can influence the prevalence of the disease [25]. The wide spread of CQ-resistant parasites prompted the WHO to recommend ACT for the management of the disease in endemic regions. In 2006, Congo changed its national policy introducing ACT for the treatment of uncomplicated malaria. The first report describing the prevalence of polymorphisms in *Pfcrt* conferring CQR showed that all the *P. falciparum* isolates were carrying the *Pfcrt* mutant alleles [2, 5]. The *Pfcrt* CVIET haplotype has been shown to be the most prevalent in Africa in contrast to the SVMNT haplotype found predominantly in Southeast Asia [23, 26]. Also, the occurrence of the *Pfcrt* M74I and N75E mutations has been linked to the K76T mutation as reported by Severini et al. who noted a 100% linkage between these alleles [27]. This explains the occurrence at the same frequency of these three mutations in this study. Studies conducted in Malawi and Tanzania reported a successful restoration of CQ-sensitive strains after the implementation of ACT for the treatment of uncomplicated falciparum malaria [28–30]. The return of these CQ-sensitive strains as observed in other studies support the hypothesis that the removal of drug pressure has led to a re-expansion of a heterogeneous susceptible parasites that might have prevailed when CQ was used.

Likewise, a significant decrease in the prevalence of the *Pfcrt* haplotypes associated with CQR was noted in this study from 2005 in republic of Congo [2], 1 year before the treatment policy was changed. However, their frequency did not decrease further after 9 years since the implementation of ACT, suggesting a possible use of CQ in the region by the population owing to the fact that it is less expensive than ACT.

Mutations in the *Pfmdr1* and changes in its copy number have been associated with the development of decreased parasite sensitivity to several anti-malarial drugs. The major SNPs in the *Pfmdr1* gene are N86Y, Y184F and D1246Y. The wild-type N86 has been shown to be responsible for the increased tolerance to drugs such as artemether [31] and 86Y associated with delayed parasite clearance with parenteral artesunate [32]. The mutant allele 86Y has been shown to associate with increased susceptibility to mefloquine in in vitro experiments [33]. Many studies that evaluated the influence of *Pfmdr1* mutations in vitro have reported that, 184F or 1042N alleles decrease artemether and lumefantrine sensitivity in Thai isolates [34], *Pfmdr1* 86Y and *Pfcrt*76T in Kenyan isolates [35]. A yet another recent study from Burkina Faso reports on influence of *Pfmdr1* 86Y and *Pfcrt*76T mutations in AL and ASAQ treatment [36]. The N86Y and Y184F mutants are prevalent in Asian and African continents while S1034C, N1042F and D1246Y alleles are common in South American countries [37]. It is interesting to note that 86Y mutant allele prevalence is decreased in 2015 when compared to 2010 cohort samples. *Pfmdr1* 86Y and 1246Y alleles are linked to CQR in Africa and the 86Y allele is associated with decreased quinine sensitivity in African clinical isolates. The fact that *Pfmdr1* gene mutations are not the principal actor in CQR [38, 39] might explain the decrease in the frequency of the *Pfmdr1* mutations linked to CQR observed between 2010 and 2015. Both *Pfmdr1* and *Pfcrt* genes are present on the membrane of the digestive vacuole in the malarial parasite and is believed to regulate the chemical solutes from the drug across the membrane. Few studies have reported that certain *Pfmdr1* and *Pfcrt* alleles are in linkage and substantiated their functional relatedness [40, 41].

The occurrence of key mutations in the *Pfatp6* gene linked to artemisinin resistance before the use of artemisinin and its derivatives in malaria treatment has been a major subject of controversy. The main question raised is whether these mutations occur as a result of some natural events or through prior exposure of parasites to drug pressure [14, 24]. One possible confounding factor could be the use of herbal plants in the treatment of malaria, which might have similar chemical constituents to artemisinin, and which is common practice in many malaria-endemic countries. Only the *Pfatp6* H243Y and E431K mutations were identified among the five key mutations investigated. The variation in the asparagine-tandem repeat region had been previously reported by Tanabe et al. [42] but this had no role in artemisinin resistance. Despite the fact that none of the driving mutations for artemisinin resistance in the *Pfk13* propeller domain reported by Ariey et al. [16] was identified, in the present study non-synonymous variants I552M and S549P are detected, which were previously reported in isolates from the Central African Republic and India, respectively.

## Conclusions

The new and unreported mutations identified in this study could help in long-term surveillance of the development of artemisinin resistance in the Republic of Congo. This study on molecular surveillance will support to predict sensitivity to different anti-malarials used in Republic of Congo and will additionally provide information on fixation of any multidrug resistance alleles in this population. The results observed in this study could serve as the basis for epidemiological studies monitoring the distribution of molecular markers of anti-malarial drug resistance in the Republic of Congo.

## Abbreviations

*Pfmdr1*: *Plasmodium falciparum* multidrug resistance 1
*Pfatp6*: *Plasmodium falciparum* Ca^2+^-ATPase
*Pfk13*: *Plasmodium falciparum* Kelch-13 propeller domain
ACT: artemisinin-based combination therapies
WHO: World Health Organization
